# SBOL Visual: A Graphical Language for Genetic Designs

**DOI:** 10.1371/journal.pbio.1002310

**Published:** 2015-12-03

**Authors:** Jacqueline Y. Quinn, Robert Sidney Cox, Aaron Adler, Jacob Beal, Swapnil Bhatia, Yizhi Cai, Joanna Chen, Kevin Clancy, Michal Galdzicki, Nathan J. Hillson, Nicolas Le Novère, Akshay J. Maheshwari, James Alastair McLaughlin, Chris J. Myers, Umesh P, Matthew Pocock, Cesar Rodriguez, Larisa Soldatova, Guy-Bart V. Stan, Neil Swainston, Anil Wipat, Herbert M. Sauro

**Affiliations:** 1 Autodesk Research, Autodesk Inc., San Francisco, California, United States of America; 2 Chemical Science and Engineering, Kobe University, Kobe, Japan; 3 Information and Knowledge Technologies, Raytheon BBN Technologies, Cambridge, Massachusetts, United States of America; 4 Electrical and Computer Engineering, Boston University, Boston, Massachusetts, United States of America; 5 School of Biological Sciences, University of Edinburgh, Edinburgh, United Kingdom; 6 Fuels Synthesis and Technologies Divisions, Joint BioEnergy Institute, Emeryville, California, United States of America; 7 Lawrence Berkeley National Lab, Berkeley, California, United States of America; 8 Synthetic Biology Unit, ThermoFisher Scientific, Carlsbad, California, United States of America; 9 Arzeda Corp, Seattle, Washington, United States of America; 10 Babraham Institute, Cambridge, United Kingdom; 11 Stanford University School of Medicine, Stanford, California, United States of America; 12 School of Computing Science, Newcastle University, Newcastle upon Tyne, United Kingdom; 13 Department of Electrical and Computer Engineering, University of Utah, Salt Lake City, Utah, United States of America; 14 Department of Computational Biology & Bioinformatics, University of Kerala, Kerala, India; 15 Turing Ate My Hamster LTD, Newcastle upon Tyne, United Kingdom; 16 Department of Biomedical Sciences, College of Medicine, Florida State University, Tallahassee, Florida, United States of America; 17 Computer Science, Brunel University, London, United Kingdom; 18 Department of Bioengineering, Centre for Synthetic Biology and Innovation, Imperial College London, South Kensington Campus, London, United Kingdom; 19 Centre for Synthetic Biology of Fine and Specialty Chemicals (SYNBIOCHEM), University of Manchester, Manchester, United Kingdom; 20 Bioengineering, University of Washington, Seattle, Washington, United States of America

## Abstract

Synthetic Biology Open Language (SBOL) Visual is a graphical standard for genetic engineering. It consists of symbols representing DNA subsequences, including regulatory elements and DNA assembly features. These symbols can be used to draw illustrations for communication and instruction, and as image assets for computer-aided design. SBOL Visual is a community standard, freely available for personal, academic, and commercial use (Creative Commons CC0 license). We provide prototypical symbol images that have been used in scientific publications and software tools. We encourage users to use and modify them freely, and to join the SBOL Visual community: http://www.sbolstandard.org/visual.

## Abstract 2

This Community Page introduces SBOL Visual—a standard visual language for DNA design and synthetic biology. Researchers are encouraged to download the symbols, use them freely, and suggest additions.

## Background

By the 1970s, molecular biologists had already developed many variations in the language used to describe functional regions of DNA, or genetic sequence *features*, with different terms used to describe similar features in different organisms. A protein-coding DNA sequence might be called a *coding sequence* (CDS), an *open reading frame*, an *exon*, or simply a *gene*, depending on the organism and method of study. To address such concerns, the *Sequence Ontology* [1] maintains a standard set of terms for describing different genetic features. This effort helped unify annotation efforts during the rise of high-throughput genome sequencing in the last decade.

Across that same decade, *synthetic biology* has advanced capabilities for forward engineering of complex genetic systems with multiple sequence features. This has increased the need for consistent terminology and representations of genetic designs. A visual representation of genetic sequence elements and their arrangement can quickly communicate adjacency, contiguity, repetition, and uniqueness. These properties become relevant as genetic designs become more complex, with multiple promoters, CDSs, etc. This is especially true for genetic designs expressed heterologously and when a system is engineered first in one organism (e.g., [2]), then moved to a different host (e.g., [3,4]).

Standards are enabling technologies for communication: standard symbols have had a profound impact in other engineering disciplines, such as the Institute of Electrical and Electronics Engineers (IEEE) standards for representing electronic components and circuits [5,6], or computer-aided design (CAD) standards for representing architecture and mechanical engineering [7,8]. Standard symbols simplify figures and user interfaces, enhance familiarity, and streamline the design process. SBOL Visual aims to have a similar salutary effect for the engineering of biological systems.

## SBOL Visual Specification

Synthetic Biology Open Language (SBOL) Visual is the product of an ongoing community effort to develop and standardize a graphical language for synthetic biology and biological engineering, focusing initially on symbols for commonly used sequence features [9]. In its current form, SBOL Visual is a set of symbols that correspond to sequence features encoded by a DNA molecule. The meaning of each symbol is established by association with terms in the Sequence Ontology (S1 and S2 Tables). SBOL Visual builds on the Sequence Ontology’s ten years of work on standardizing precise definitions of genetic sequence features, and the success of this work ensures that SBOL Visual symbols are well aligned with established scientific vocabulary. The mapping to Sequence Ontology terms also connects SBOL Visual to the SBOL data exchange standard, enabling automatic mapping from data to a graphical representation [9].

Though SBOL Visual makes use of Sequence Ontology terms, the two projects address objectives of differing scope. The Sequence Ontology provides a controlled vocabulary for all functional genetic features, while SBOL Visual focuses on facilitating the communication of engineered genetic designs. One driving need for SBOL Visual is to abstract and simplify the full complexity of sequence features that may be represented with a single symbol, e.g., the promoter, which is composed of many functional subsequences [10]. For example, the “bent arrow” symbol used by SBOL Visual for a promoter (SO:0000167) could also be used to denote the transcription start (SO:0000315) when describing the substructure of the same promoter. Such usage is common in the scientific literature, and unambiguous since the descriptions are at different levels of detail. As such, each SBOL Visual symbol can map to one or more terms in the Sequence Ontology. For features common in engineered systems but not yet in the Sequence Ontology, SBOL Visual has contributed back to the ongoing development of Sequence Ontology by contributing several new terms (e.g., restriction_enzyme_assembly_scar, SO:0001953).

In developing these symbols, the community initially selected a set of commonly used genetic parts and a set of features relevant for the assembly of DNA molecules. The symbols were chosen based on common depiction of genetic designs in molecular, systems, and synthetic biology publications. The 12 core features (S1 Table) include typical sequences needed for the proper functioning of a gene: such as signals for DNA replication, RNA transcription, and protein translation. We specify the simple rectangle symbol to be a “user-defined” catchall for special genetic features not currently part of SBOL Visual. We also include nine symbols to describe common methods of DNA assembly, such as restriction sites (S2 Table). Together these 21 symbols were proposed and ratified as SBOL Visual 1.0 Specification.

SBOL Visual was designed to serve a range of formats, including whiteboard discussions, slide presentations, scientific publications, and *computer-aided design* [11]. Some symbols were adjusted to account for communication goals, particularly to increase visual distinctiveness, to decrease feature orientation ambiguity, and to facilitate rapid design. The SBOL Visual symbols can thus be drawn quickly by hand, used as stencil art with computer illustration software, rendered in a web browser, used as image primitives for building software tools, or applied as a formal symbology for communication and instruction. Inspired by the clarity, simplicity, and usefulness of electronic symbols, stylistic features such as scaling, line-width, color, and use of text labels are left explicitly unspecified in SBOL Visual. This built-in flexibility facilitates variation in style for functional or aesthetic purposes, which allows genetic designers and software developers to differentiate their images and interfaces to express additional information (e.g., module structure, protein type, origin of components) or artistic style while still benefiting from the standard. Thus, SBOL Visual provides a set of readily distinguishable basic shapes, which can be refined for individual applications.

## Current Use in Publications and Software

**Fig 1:**
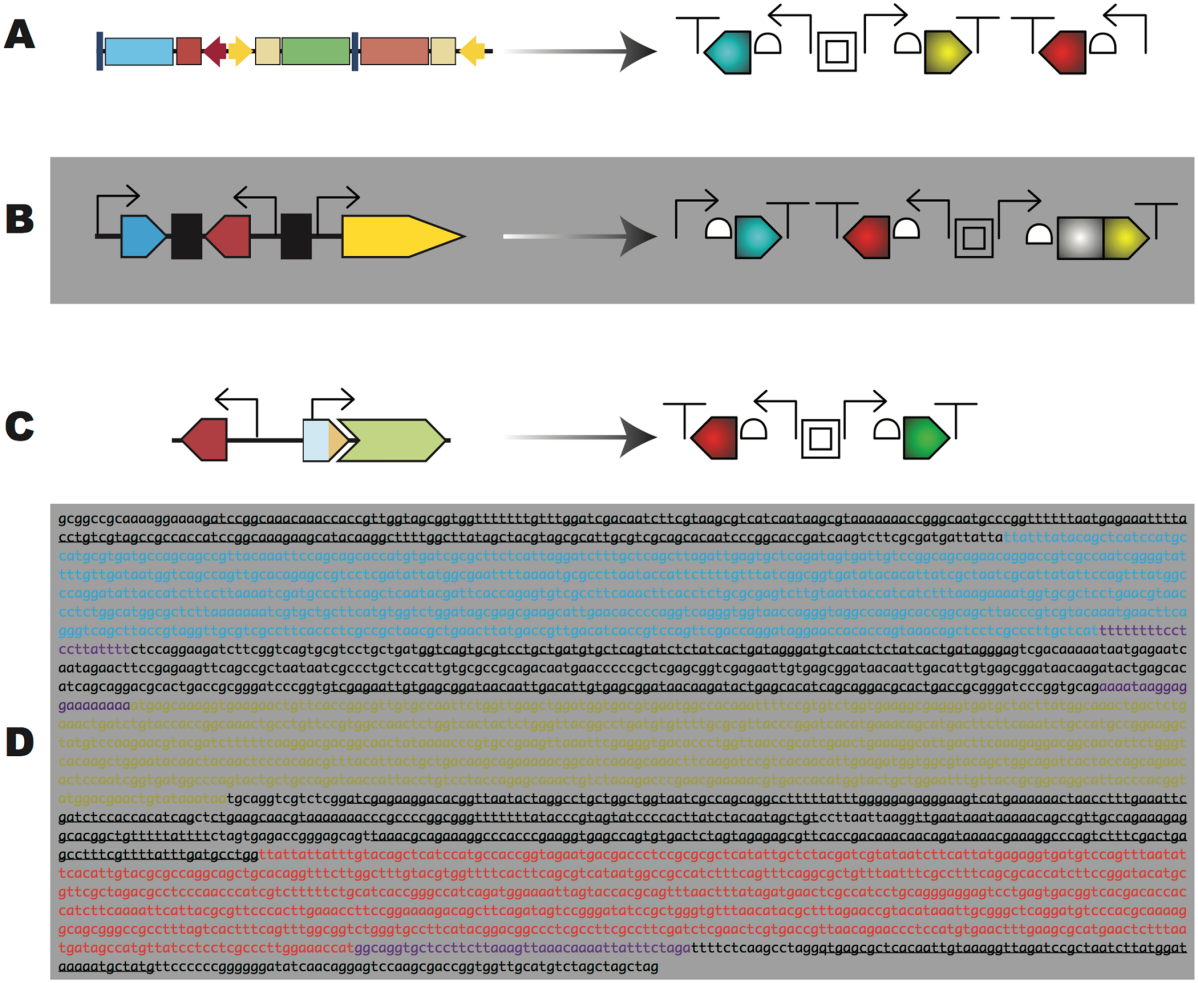
SBOL Visual aids rapid communication of variant synthetic DNA designs: multi-color reporter variants. In this figure we present an example genetic reporter device [17,21] to show how a common symbology can highlight the differences between a series of related DNA constructs. (A) The three-color, green fluorescent protein (GFP)-based genetic reporter was designed for easy swapping of each cassette’s promoter by methods based on restriction cloning or recombination, allowing it to be modified to measure promoters for analyzing the effects of growth condition on circuit performance variation (figure modified from [20]). The SBOL Visual symbology (right) highlights that the promoters are variable, and the coloring of the CDS (which is not constrained by SBOL Visual) is used to keep track of which genetic reporter is at which position, including three fluorescent proteins (CFP: cyan, YFP: yellow, RFP: red). Equivalent means for distinguishing sequences include fill/hatch patterns (e.g., for black-and-white publication) or textual labels above the components. (B) The three-color reporter was modified to make a protein fusion between the Yellow Fluorescent Protein gene and the cI repressor from phage lambda (modified from [21] gray and yellow in the SBOL Visual diagram). This shows how the user-specified coloring can add new information to the basic glyphs. This design also swapped around the positions of the different reporter genes, as well as replacing the promoters, to create a regulatory circuit in which the regulatory protein could be directly observed via the protein fusion with YFP. (C) The three-color reporter was modified to a two-color system, and the YFP gene was replaced with a GFP variant, to optimize two-color measurement. This system was used to systematically measure all combinations of 114 promoters and 111 ribosome binding sites (modified from [18]). (D) The DNA sequence of the three-color reporter construct presented in [17]. Here the promoter and terminator sequences are underlined, and the three color fluorescent genes are indicated by highlighting the text as cyan, yellow, or red. The purple sequences denote ribosome binding sites. This highlights how the SBOL Visual notation in (A) is much easier to quickly understand than the raw sequence, while still clearly communicating the organization of genetic parts.

Several scientific publications have already adopted SBOL Visual (e.g., [12–16]). To illustrate the potential benefits of SBOL Visual in publication, consider the development of the multicolor genetic reporter system shown in Fig 1. This system was initially built with three fluorescent protein genes to measure genetic regulation and noise in *Escherichia coli* [17], and it was later modified to use as a test bed for measuring combinations of promoters and ribosome binding sites [18], the effect of codon bias on the initiation of translation [19], and to analyze the effects of carbon and nitrogen metabolism on synthetic circuit performance [20]. When originally published, each of these publications used modified forms of the genetic system and slightly different symbols to depict the genetic features (Fig 1A–1C left-side images). Unifying such variant genetic visual depictions with a common symbology (Fig 1A–1C right-side images) makes their relationship much more immediately apparent, and clearly communicates their structural differences. We expect the use of SBOL Visual to disseminate new results, inspire new genetic designs, and communicate ideas across human language barriers.

Adoption of SBOL Visual by software tools (see Box 1) has further helped to drive use of SBOL Visual in publication. For example, early versions of the SBOL Visual symbols in Clotho Spectacles [22] were used to produce schematics for a genetic system able to convert ambient nitrogen to ammonia (nitrogen fixation) (S1 Fig) [13]. The SBOL Visual symbols have also been used to represent software-generated combinatorial designs of transcriptional cascades and feed-forward circuits [23], and in layered transcriptional circuit devices derived from CRISPR for use in mammalian cells [15]. In sum, SBOL Visual is becoming an accepted standard for communicating genetic designs, both in software and in the scientific literature.

**Box 1. SBOL Visual Adoption in Academic and Commercial Software Tools Enables Clear Design Specification and Accurate Sequence Annotation**

- Pigeon [24] is a synthetic biology design visualizer that generates SBOL Visual figures from terse strings similar to classical genotype notation with a great deal of flexibility, including the ability to color-code, invert, and add text descriptions to the symbols.
- The ICE genetic design repository software platform [25] includes an SBOL Visual view (created automatically using Pigeon) with DNA component types (CDS, terminator, etc.), encouraging more accurate sequence annotation. This view is automatically generated from the uploaded annotated sequence file.
- The GraphViz graph visualization package [26] includes SBOL visual symbols, which can be used to visualize genetic constructs and include them as components in arbitrary graph diagrams.
- SBOL Visual Web Widgets (see Table 1) allow for dynamically rendering the symbols in Scalable Vector Graphics (SVG) and their styling using Cascading Style Sheets (CSS). The current set of available widgets include CDS, promoter, terminator, and restriction site.
- The VisBOL Web-based design visualizer (see Table 1) is an open-source tool enabling the dynamic, automated generation of SBOL Visual designs from SBOL documents in the browser, while DasBOL (see Table 1) provides a web service for querying relationships between SBOL Visual and Sequence Ontology terms.
- SBOL Visual symbols are used as interactive abstractions of genetic parts in a number of genetic design applications, including BioCompiler [16], Clotho [22], DeviceEditor [27], GenoCAD [28], SBOL Designer [29] and its Geneious extension [30], TeselaGen Design Editor [31], Tinkercell [32], and VectorNTI Express Designer [33].

**Table 1:** Links to SBOL Visual resources and related tools.

**SBOL Visual website**: Project page with information about SBOL Visual and download links	http://sbolstandard.org/visual/
**Sequence Ontology**: Terms used to establish SBOL Visual symbol meanings	http://sequenceontology.org/
**Pigeon**: Web tool for fast and easy scripting of SBOL Visual diagrams (open public web server)	http://pigeoncad.org/
**ICE Genetic Repository**: Repository for parts, generates SBOL Visual from annotated sequence files (open source but requires login account to use public web server)	https://public-registry.jbei.org
**Web Widgets**: JavaScript SBOL Visual web renderer (open source with live demo)	http://drdozer.github.io/sbolv/
**VisBOL**: Open-source web-based SBOL Visual design visualizer in JavaScript, with support for visualizing Genbank and SBOL standard file formats	http://visbol.org/design/
**DasBOL**: A web service which looks up SBOL Visual terms associated with Sequence Ontology codes	http://dasbol.org/
**DeviceEditor**: A visual biological CAD canvas (not open source and requires registration)	https://j5.jbei.org/
**TeselaGen Design Editor:** A visual biological CAD canvas (commercial software, requires registration, free for academic use with registration)	https://teselagen.com/

## New Symbol Adoption and Symbol Variations Process

Although SBOL Visual can express many useful features of genetic designs, it is not complete. SBOL Visual is developed through an open and ongoing community process, and any synthetic biology practitioner is encouraged to propose new symbols and modifications to existing symbols. This is done by submitting a proposal to the SBOL Visual working group by email, containing the proposed name, symbol, associated Sequence Ontology term, and motivation for addition of the symbol. In cases in which no appropriate Sequence Ontology term exists, the SBOL Visual working group requests one; we have so far submitted ten such terms to the Sequence Ontology. The proposed symbol then goes through an endorsement process, a trial period, and finally adoption by open community vote.

Using the same process, a symbol may be proposed as a *variant* of an existing symbol. Symbol variants share the same Sequence Ontology term, but may be more useful in different contexts. For example, an asymmetric variant of the symbol for transcriptional terminator has been proposed in order to better capture the often directional behavior of these genetic parts. While a more complex shape, it allows for more explicit specification of directionality when significant. Variant symbols may also be proposed by software tools, to allow for style differences between user interfaces. Variants also provide a path for improvement of symbols and deprecation of obsolete versions over time, and they allow contextual details and styles to be recognized by the SBOL Visual standard.

## Collaboration and Future Work

SBOL Visual can be combined with other graphical languages, such as symbols from the *Systems Biology Graphical Notation* (SBGN), a standard for depiction of biological regulatory networks and molecular interactions [34]. SBOL and SBGN are both core standards of the COMBINE community project [35]. The SBGN Activity Flow language has codified the common use of network interactions diagrams between biological components, and can display regulatory interactions between genetic parts represented by SBOL Visual (S1A Fig). The SBOL Visual and SBGN development groups are collaborating with the aim of enabling seamless and unambiguous use of both standards. With such collaborations and the development of further SBOL Visual symbols, we hope to enable better depiction at the various levels of organization required to represent engineered biological systems.

Parallel efforts are underway to extend the language with higher-level concepts like genetic devices, genetic systems, and cellular chassis. At the sequence level, many genetic features become strongly dependent on the host organism. We envision that several of the 12 core design SBOL Visual symbols could be expanded in detail to more detailed visual languages.

For example, the CDS symbol could display more detailed information about the protein it encodes. For rational protein engineering, a common design task is to fuse two different protein functional domains into a single molecule, such as the repressor-YFP fusion depicted Fig 1B. Often, designs use a flexible linker sequence in between two functional domains. One future goal is to develop a systematic visual representation of protein coding sequences that can display the design choices in making such fusions. This protein design language could also include distinctive symbols representing design elements such as cellular localization signals, protein degradation signals, phosphorylation sites, protein cleavage sites, and purification tags. Many functional protein domains have been annotated by SwissProt and UniProtKB [36] and provide a rich source of sequences for rational protein design. For example, an inducible eukaryotic transcription factor was rationally designed by fusing domains for DNA binding, estrogen response, and promoter activation [37]. We hope to extend SBOL Visual to be able to visually depict such feats of engineering at the sequence design level.

## The SBOL Visual Website and Data Distribution

The SBOL Visual website hosts prototypical symbol images in several formats, including vector files, an Omnigraffle stencil set, a set of web widgets, and a TrueType SBOL Visual font for word processors. The website also provides human-readable mappings between our terms and the Sequence Ontology terms, descriptions of how these terms are being used in CAD tools, and instructions for submitting new symbols and symbol variants. A machine-readable mapping of SBOL Visual symbols to common GenBank feature keys, Sequence Ontology terms, and Pigeon and Graphviz codes are provided as S1 Data and are also available via the SBOL Visual website. The SBOL Visual specification is published as a BioBrick Foundation Request for Comments (BBF RFC) upon approval by the SBOL Developers Group. SBOL Visual version 1.0.0, was published as BBF RFC 93 [38]. To make suggestions, ask questions, or join the SBOL Visual working group, visit the SBOL Visual website or email visual@sbolstandard.org.

## Supporting Information

S1 Data Machine-readable mapping of SBOL Visual symbols to common GenBank feature keys, Sequence Ontology terms, and Pigeon and Graphviz codes in CSV format. (CSV)

S1 Fig SBOL Visual diagrams can be created by multiple methods for various use cases and levels of formality. A gene regulatory network for production of T7 RNA Polymerase as an output to isopropyl-beta-D-thiogalactopyranoside(IPTG) AND aTc logic as presented by Temme et al. is shown: (A) informally sketched on a whiteboard, as might be done in design brainstorming, and (B) generated using Pigeon. These illustrate how different stages of the design process use the symbols differently. For example, (A) and (B) include regulation arcs while the figure in the original manuscript includes origin of replication and resistance markers. Also illustrated here is how symbol use influences the development of the standard. Use of the circle around an “x” for spacer by Temme et al. led to the inclusion of the symbol in Pigeon, and the symbol is currently going through the new symbol adoption process. Parts (A) and (B) demonstrate depiction of regulation alongside SBOL Visual. For example, “*repressor tetR* represses *promoter Ptet*”is represented by a line linking the tetR CDS symbol with the Ptet promoter symbol. This is formally described by SBGN Activity Flow as an *inhibitory arc*. In such combined diagrams, SBOL Visual depicts the genetic parts while SBGN depicts the network of interactions between biological components. SBGN is particularly compatible with SBOL Visual, and the vast majority of regulation maps in synthetic biology publications can be described using the SBGN Activity Flow language. In Activity Flow, nodes represent “activities” (e.g., gene activity) while arcs represent the effect of one activity on another. Therefore, SBOL Visual symbols can represent Activity Flow nodes, with arcs representing the regulation logic.
(TIFF)

S1 Table SBOL Visual symbols for genetic design. (PDF)

S2 Table SBOL Visual symbols used for cloning and sequencing. (PDF)

## Abbreviations

BBF RFC: BioBrick Foundation Request for Comments
CAD: computer aided design
CDS: coding sequence
CFP: cyan fluorescent protein
CSS: Cascading Style Sheets
GFP: green fluorescent protein
IEEE: Institute of Electrical and Electronics Engineers
IPTG: isopropyl-beta-D-thiogalactopyranoside
RFP: red fluorescent protein
SBOL: Synthetic Biology Open Language
SBGN: Systems Biology Graphical Notation
SVG: Scalable Vector Graphics
YFP: yellow fluorescent protein
