# Supplementary figures and images for: SBOL Visual: A Graphical Language for Genetic Designs
Source: PLoS Biol. 2015 Dec 3;13(12):e1002310. doi: 10.1371/journal.pbio.1002310 (PMC4669170; doi:10.1371/journal.pbio.1002310)

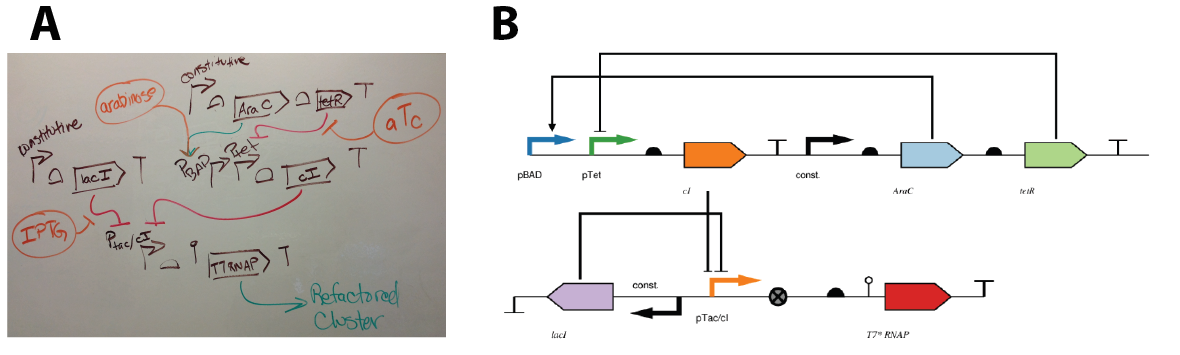

Supplement: S1 Fig — A gene regulatory network for production of T7 RNA Polymerase as an output to isopropyl-beta-D-thiogalactopyranoside(IPTG) AND aTc logic as presented by Temme et al. is shown: (A) informally sketched on a whiteboard, as might be done in design brainstorming, and (B) generated using Pigeon. These illustrate how different stages of the design process use the symbols differently. For example, (A) and (B) include regulation arcs while the figure in the original manuscript includes origin of replication and resistance markers. Also illustrated here is how symbol use influences the development of the standard. Use of the circle around an “x” for spacer by Temme et al. led to the inclusion of the symbol in Pigeon, and the symbol is currently going through the new symbol adoption process. Parts (A) and (B) demonstrate depiction of regulation alongside SBOL Visual. For example, “repressor tetR represses promoter Ptet”is represented by a line linking the tetR CDS symbol with the Ptet promoter symbol. This is formally described by SBGN Activity Flow as an inhibitory arc. In such combined diagrams, SBOL Visual depicts the genetic parts while SBGN depicts the network of interactions between biological components. SBGN is particularly compatible with SBOL Visual, and the vast majority of regulation maps in synthetic biology publications can be described using the SBGN Activity Flow language. In Activity Flow, nodes represent “activities” (e.g., gene activity) while arcs represent the effect of one activity on another. Therefore, SBOL Visual symbols can represent Activity Flow nodes, with arcs representing the regulation logic. (TIFF) [file pbio.1002310.s002.tiff]
