# Supplementary material for: SBOL Visual: A Graphical Language for Genetic Designs
Source: PLoS Biol. 2015 Dec 3;13(12):e1002310. doi: 10.1371/journal.pbio.1002310 (PMC4669170; doi:10.1371/journal.pbio.1002310)
Supplement: S1 Table — (PDF) [file pbio.1002310.s003.pdf]

Supplementary Table 1. SBOL Visual Symbols

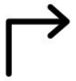	**Promoter**<br>*The sequence at the beginning of a gene required for transcription initiation.*<br>SO Accession: SO:0000167	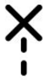	**Ribonuclease Site**<br>*A region of a transcript encoding the cleavage site for a ribonuclease enzyme.*<br>SO Accession: SO:0001977
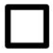	**Operator**<br>*A transcription factor binding site.*<br>SO Accession: SO:0000057	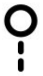	**RNA Stability Element**<br>*A variant that changes the stability of a transcript with respect to a reference sequence.*<br>SO Accession: SO:0001546
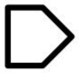	**Coding Sequence (CDS)**<br>*A complete coding sequence, inclusive of the start and stop codons. A sequence of codons which encode an oligopeptide, often folds into a protein. May also include untranslated regions (e.g. introns).*<br>SO Accession: SO:0000316	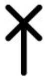	**Protease Site**<br>*A polypeptide_region that codes for a protease cleavage site.*<br>SO Accession: SO:0001956
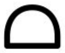	**Ribsome Entry Site**<br>*This symbol represents a ribosome binding site in prokaryotes, an IRES in eukaryotic viruses, and Kozak sequence in eukaryotes.*<br>SO Accession: SO:0000139	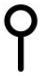	**Protein Stability Element**<br>*A polypeptide region that proves structure in a protein that affects the stability of the protein.*<br>SO Accession: SO:0001955
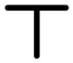	**Terminator**<br>*A sequence which halts transcription, usually found at the end of a gene.*<br>SO Accession: SO:0000141	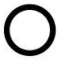	**Origin of Replication**<br>*The origin of replication; starting site for duplication of a nucleic acid molecule to give two identical copies.*<br>SO Accession: SO:0000296
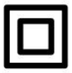	**Insulator / Spacer**<br>*A sequence that provides genetic isolation between two or more contiguous components.*<br>SO Accession: SO:0000627	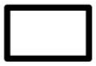	**User Defined**<br>*A region of a DNA sequence not covered by other SBOL Visual symbols.*<br>SO Accession: N/A
