# Supplementary material for: SBOL Visual: A Graphical Language for Genetic Designs
Source: PLoS Biol. 2015 Dec 3;13(12):e1002310. doi: 10.1371/journal.pbio.1002310 (PMC4669170; doi:10.1371/journal.pbio.1002310)
Supplement: S2 Table — (PDF) [file pbio.1002310.s004.pdf]

Supplementary Table 2: DNA Construction Symbols

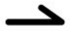	**Primer Binding Site**<br>*Non-covalent primer binding site for initiation of replication, transcription, or reverse transcription.*<br>SO Accession: SO:0005850	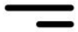	**5' Overhang**<br>*A terminal region of DNA sequence where the end of the region is not blunt ended and the exposed single strand terminates at the 5' end.*<br>SO Accession: SO:0001932
	**Restriction Enzyme Recognition Site**<br>*A binding site that, in the nucleotide molecule, interacts selectively and noncovalently with polypeptide residues of a restriction enzyme.*<br>SO Accession: SO:0000061, SO:0001687	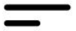	**3' Overhang**<br>*A terminal region of DNA sequence where the end of the region is not blunt ended and the exposed single strand terminates at the 3' end.*<br>SO Accession: SO:0001933
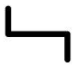	**5' Sticky restriction site**<br>*A restriction enzyme recognition site that, when cleaved, results in 5 prime overhangs.*<br>SO Accession: SO:0001975	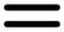	**Assembly Junction / Assembly Scar**<br>*A region of DNA sequence formed from the ligation of two sticky ends where the palindrome is broken and no longer comprises the recognition site and thus cannot be re-cut by the restriction enzymes used to create the sticky ends.*<br>SO Accession: SO:0001953
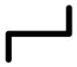	**3' Sticky restriction site**<br>*A restriction enzyme recognition site that, when cleaved, results in 3 prime overhangs.*<br>SO Accession: SO:0001976	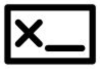	**Signature / Barcode**<br>*A region of sequence where developer information is encoded.*<br>SO Accession: SO:0001978
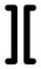	**Restriction Site Resulting in No Overhang /**<br>**Blunt Restriction Site**<br>*A restriction enzyme recognition site that, when cleaved, results in no overhangs.*<br>SO Accession: SO:0001691		
